# Effectiveness of BNT162b2 and ChAdOx-1 vaccines in residents of long-term care facilities in England using a time-varying proportional hazards model

**DOI:** 10.1093/ageing/afac115

**Published:** 2022-05-20

**Authors:** Karthik Paranthaman, Sathyavani Subbarao, Nick Andrews, Freja Kirsebom, Charlotte Gower, Jamie Lopez-Bernal, Mary Ramsay, Andrew Copas

**Affiliations:** Field Service, UK Health Security Agency (UKHSA), London SW1P 3JR, UK; Immunisation and Countermeasures Division, UK Health Security Agency (UKHSA), London NW9 5EQ, UK; Immunisation and Countermeasures Division, UK Health Security Agency (UKHSA), London NW9 5EQ, UK; Immunisation and Countermeasures Division, UK Health Security Agency (UKHSA), London NW9 5EQ, UK; Immunisation and Countermeasures Division, UK Health Security Agency (UKHSA), London NW9 5EQ, UK; Immunisation and Countermeasures Division, UK Health Security Agency (UKHSA), London NW9 5EQ, UK; Immunisation and Countermeasures Division, UK Health Security Agency (UKHSA), London NW9 5EQ, UK; Institute for Global Health, University College London, London, UK

**Keywords:** Covid-19, older people, vaccine effectiveness

## Abstract

**Introduction:**

residents of long-term care facilities (LTCFs) are at high risk of adverse outcomes from SARS-CoV-2. We aimed to estimate the vaccine effectiveness (VE) of one and two doses of BNT162b2 and ChAdOx-1 against SARS CoV-2 infection and COVID-19-related death in residents of LTCFs.

**Methods:**

this observational study used testing, vaccination and mortality data for LTCF residents aged ≥ 65 years who were regularly tested regardless of symptoms from 8 December 2020 to 30 September 2021 in England. Adjusted VE, calculated as one minus adjusted hazard ratio, was estimated using time-varying Cox proportional hazards models for infection and death within 28 days of positive test result. Vaccine status was defined by receipt of one or two doses of vaccine and assessed over a range of intervals.

**Results:**

of 197,885 LTCF residents, 47,087 (23.8%) had a positive test and 11,329 (5.8%) died within 28 days of a positive test during the study period. Relative to unvaccinated individuals, VE for infection was highest for ChAdOx-1 at 61% (40–74%) at 1–4 weeks and for BNT162b2 at 69% (52–80%) at 11–15 weeks following the second dose. Against death, VE was highest for ChAdOx-1 at 83% (58–94%) at 1–4 weeks and for BNT162b2 at 91% (75–97%) at 11–15 weeks following second dose.

**Conclusions:**

compared with unvaccinated residents, vaccination with one dose of BNT162b2 or ChAdOx-1 provided moderate protection against infection and death in residents of LTCFs. Protection against death improved after two doses. However, some waning of protection over time was noted.

## Key Points

- Receipt of either ChAdOx-1 or BNT162b2 offers modest protective effect against infection.
- After 4 weeks from receipt of second dose, either vaccine offers over 80% protection against death.
- Some waning of protection over time was noted.

## Introduction

Across the world, severe outcomes due to SARS-CoV-2 have disproportionately affected residents of long-term care facilities (LTCFs). By 2 April 2021, there had been 173,974 deaths involving COVID-19 among LTCF residents in England and Wales [1]. To date, multiple vaccines have been developed and approved for use [2, 3]. Rollout of the Covid-19 vaccination programme began on 8 December 2020 in the UK initially with BNT162b2 mRNA vaccine, followed by ChAdOx-1 adenoviral vector vaccine in January 2021. LTCF residents and staff were given priority for vaccination in the UK. The vaccination programme was initially implemented with the second dose offered 3 weeks following the first dose. Following recognition that the Alpha variant was spreading rapidly, an extended interval of 12 weeks between first dose and second dose implemented in January 2021 [4]. The primary aim of this change was to maximise the proportion of those most at risk receiving their first vaccine dose early to reduce hospitalisations and deaths. This meant that LTCF residents who received their first dose in the first 4–6 weeks of the vaccination programme received their second dose after 3 weeks, whereas those due their second dose after the change in UK policy received their second dose around 12 weeks from first dose. A further booster (third) dose of vaccine was offered to LTCF residents from 16 September 2021.

Although real-world vaccine effectiveness (VE) data are emerging from several settings, given the higher risk for older adults and immunosenescence [5], it is important to focus on VE in this age group. A study in the UK on adults older than 70 years found that VE against symptomatic infection was 61% (95% confidence interval [CI], 51–69%) 28–34 days after a single dose of BNT162b2 and 73% (95%CI, 27–90%) from Day 35 onwards with ChAdOx-1 [6]. A study in Israel among adults aged over 85 years showed VE after two doses of BNT162b2 against infection was 94.2% (95%CI, 91.9–95.7%) and hospitalisations was 97.4% (95%CI, 95.9–98.3%) [7]. Another study in Spain found that two doses BNT162b2 were 97.0% (95%CI, 91.7–98.9%) effective in preventing COVID-19 deaths in residents of LTCFs [8].

The most recent official data from 2011 showed that there were around 291,000 people aged 65 years and above resident in LTCFs in England [9]. Residents of LTCFs in England have been offered routine testing for SARS-CoV-2 monthly since July 2020 regardless of symptoms and have access to testing if they develop symptoms consistent with COVID-19.

Given limited data on VE following two vaccine doses in LTCF residents, the primary aim of this study was to estimate the effectiveness of one and two doses of the COVID-19 vaccines against SARS CoV-2 infection and COVID-19-related death in LTCF residents across England.

## Methods

### Study design, period and setting

In this observational population study, we analysed surveillance data from the study period 8 December 2020 to 30 September 2021. The study population were residents greater than 65 years in LTCFs in England with at least two recorded tests for SARS CoV-2 and at least one test during the study period. Residents in LTCFs were identified based on their National Health Service (NHS) number and unique property reference number or postcode for those aged over 65 years. In the primary analysis, all residents with a positive test prior to 8 December 2020 were excluded. VE studies are undertaken by the UK Health Security Agency as part of ongoing surveillance activities and did not require ethical approval.

### Data sources and linkage

Data on all test results (negative and positive) from lateral flow device (LFD) and polymerase chain reaction (PCR) testing between 8 December 2020 and 30 September 2021 were extracted. Individual vaccination records in national immunisation management system database, a comprehensive database of all COVID-19 immunisations in England, were linked to testing data using NHS number, date of birth, first name and surname and postcode. Data on all-cause death and date of death for all individuals in the study were sourced from the Office for National Statistics. Weekly SARS-CoV-2 incidence rate per 100,000 population were calculated at the Local Authority level and linked to individuals based on postcode.

### Outcomes and exposures

Primary outcomes were PCR or LFD confirmed SARS-CoV-2 infection (whether symptomatic or asymptomatic) in the study period and COVID-related death. The UK definition of COVID-related death is all-cause death occurring within 28 days of a recorded positive test in the study period [10].

Individuals with a recorded test result prior to study start date entered the study on 8 December 2020. Other individuals without a previous test entered the study on their first test date during the study period. The key exposure was vaccination status by vaccine type, specifically a time-varying indicator of the time from receipt of each dose. Each individual’s vaccination status (unvaccinated, 1 dose or 2 doses) and dates were used to create time variables at risk through the study period.

For the first dose-related time periods, individuals entered the risk period on the date of receipt of first vaccine and were censored at the date of their positive test or last test or date of receipt of second dose, whichever was earliest. For the second dose-related time periods, individuals entered the risk period on the date of receipt of second dose and were censored at the earliest of date of positive test or last test or receipt of third dose of vaccine. The first dose-related time periods were 1–2, 3, 4, 5, 6–7, 8–10 and 11+ weeks after dose for infection outcome and 1–2, 3–4, 4–8, 9+ weeks after dose for death outcome. For both outcomes, the second dose-related time periods were 1–4, 5–10, 11–15, 16–20, 21+ weeks after dose. Although testing data were censored at 30 September 2021, we extended death data to 30 November 2021 to allow for deaths within 28 days of a positive test and reporting delays. Covariates included sex, age-group (in 5-year age bands, starting from 65 years), relative deprivation and 7-day moving incidence rate at Local Authority level updated daily.

### Statistical analysis

Cox proportional hazards models were used to derive adjusted hazard ratios (aHRs) with 95% CIs for the risk of infection and COVID-related death in each time period following vaccination compared with those who were unvaccinated. To account for similarities between individuals in the same care home, we included a random cluster term for care home postcode in all models. Against the main outcome measures of infection and death, aHRs are presented by vaccine type and for either vaccine, with the latter intended to provide a single estimate of effectiveness given the similarities in effect for both vaccine types. VE was calculated as (1−aHR)×100.

In post hoc analysis, we tested for evidence of waning of protection for second dose by refitting the models with revised time periods of 1–4, 5–10, 10–15, 16+ weeks after dose. For infection and death as outcome, we compared the time period with the lowest aHR for second dose against 16+ weeks for each vaccine type by changing the reference category as appropriate. To explore the effect of interval between first and second dose, we ran additional models with dosing interval as a linear predictor for time periods following receipt of second dose, after ‘centering’ by subtracting the median dosing interval (10 weeks for both vaccines). We hypothesised that the effect of dosing interval might have different effects in the immediate period (1–4 weeks) and later period (>4 weeks) after second dose, because the former would include ongoing effects of the first dose, and included separate terms for interval by vaccine manufacturer. In another model, we estimated aHRs on the risk of infection for individuals recorded as having had a positive test >90 days prior to 8 December 2020. Finally, we also conducted subgroup analysis to separate the effects on individuals living in residential and nursing LTCFs. Further details on methods and additional data are provided in Supplementary material.

## Results

**Figure 1:**
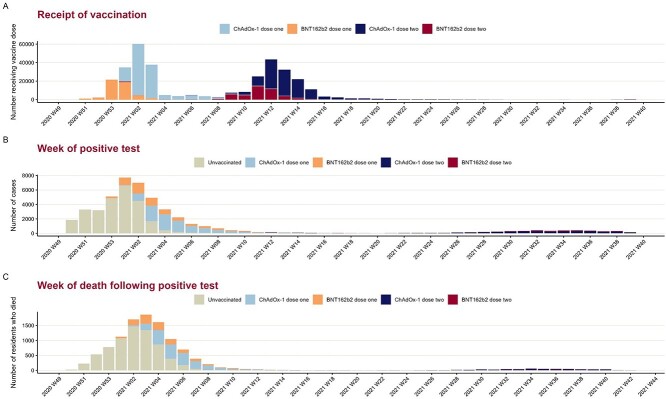
Distribution of vaccine receipt, positive test and death in LTCF residents, England.

The vaccination programme in England started with BNT162b2 on 8 December 2020 with ChAdOx-1 becoming the primary main vaccine type from January 2021 (Figure 1). A small number of residents received their second dose of BNT162b2 vaccine 3 weeks after their dose in early January. However, the vast majority received their second dose 8–12 weeks after the first dose. The median interval between first and second dose was 10 weeks for both vaccine recipients (Supplementary Figure S1).

Overall, 216,473 individuals were classified as LTCF residents, among which 185,88 (8.6%) had a previous positive test and were removed from the primary analysis. Among the remaining 197,885 individuals, 17,649 (8.9%) were unvaccinated, 16,885 (8.5%) had received one dose of vaccine and the rest 163,351 (82.5%) received two doses of vaccine by the end of the study period.

Characteristics of individuals by the number of doses and type of vaccine received at the end of time at risk during study period are shown in Table 1. Characteristics were similar across groups except that unvaccinated individuals were most likely to have had a positive test in the study period. The difference in the number of tests in the study period is a consequence of the length of time individuals were at risk.

**Table 1:** Characteristics of LTCF residents by vaccine dose received at the end of time at risk for infection

Variable	Levels	Unvaccinated^**a**^	ChAdOx-1 one dose^**a**^	BNT162b2 one dose^**a**^	ChAdOx-1 two doses^**a**^	BNT162b2 two doses^**a**^	Total^**a**^
Total		35,742 (18.1)	21,669 (11.0)	11,499 (5.8)	89,747 (45.4)	39,228 (19.8)	197,885
Age group	65–69 years	1,480 (4.1)	846 (3.9)	385 (3.3)	5,057 (5.6)	1,637 (4.2)	9,405 (4.8)
	70–74 years	2,864 (8.0)	1,527 (7.0)	791 (6.9)	8,089 (9.0)	3,107 (7.9)	16,378 (8.3)
	75–79 years	4,311 (12.1)	2,470 (11.4)	1,291 (11.2)	11,105 (12.4)	4,904 (12.5)	24,081 (12.2)
	80–84 years	6,832 (19.1)	4,013 (18.5)	2,158 (18.8)	16,763 (18.7)	7,614 (19.4)	37,380 (18.9)
	85–89 years	8,975 (25.1)	5,427 (25.0)	2,940 (25.6)	21,715 (24.2)	9,960 (25.4)	49,017 (24.8)
	90+ years	11,280 (31.6)	7,386 (34.1)	3,934 (34.2)	27,018 (30.1)	12,006 (30.6)	61,624 (31.1)
Sex	Female	23,994 (67.1)	14,924 (68.9)	7,775 (67.6)	64,094 (71.4)	28,148 (71.8)	138,935 (70.2)
	Male	11,693 (32.7)	6,684 (30.8)	3,695 (32.1)	25,542 (28.5)	11,035 (28.1)	58,649 (29.6)
	(Missing)	55 (0.2)	61 (0.3)	29 (0.3)	111 (0.1)	45 (0.1)	301 (0.2)
Relative deprivation	1 (least deprived)	5,984 (16.7)	3,850 (17.8)	2,042 (17.8)	15,635 (17.4)	7,251 (18.5)	34,762 (17.6)
	2	7,272 (20.3)	4,460 (20.6)	1,947 (16.9)	18,394 (20.5)	6,835 (17.4)	38,908 (19.7)
	3	7,635 (21.4)	4,769 (22.0)	2,410 (21.0)	19,120 (21.3)	8,073 (20.6)	42,007 (21.2)
	4	7,550 (21.1)	4,517 (20.8)	2,646 (23.0)	20,065 (22.4)	8,419 (21.5)	43,197 (21.8)
	5 (most deprived)	7,243 (20.3)	4,024 (18.6)	2,432 (21.1)	16,452 (18.3)	8,602 (21.9)	38,753 (19.6)
	(Missing)	58 (0.2)	49 (0.2)	22 (0.2)	81 (0.1)	48 (0.1)	258 (0.1)
Median number of tests in study period (IQR)		2.0 (1.0–3.0)	4.0 (2.0–6.0)	3.0 (2.0–5.0)	11.0 (9.0–14.0)	11.0 (9.0–14.0)	9.0 (3.0–13.0)
Positive test result in study period	No	8,854 (24.8)	11,901 (54.9)	5,371 (46.7)	86,592 (96.5)	38,080 (97.1)	150,798 (76.2)
	Yes	26,888 (75.2)	9,768 (45.1)	6,128 (53.3)	3,155 (3.5)	1,148 (2.9)	47,087 (23.8)

^a^Values are counts (percentages in parenthesis) except for median number of tests IQR: interquartile range

Among 197,885 individuals, 752 individuals (<0.01%) were missing information on any covariate. Of the remaining 197,133 individuals, 91.7% (178,500) entered the study on 8 December 2020 and the remaining 8.3% (18,633) joined the study at a later date. The distribution of follow-up time for individuals in the analysis for infection as outcome is given in Supplementary Figure S2. Although community incidence rates were incorporated in the models at the Local Authority level, Supplementary Figure S3 provides an overview of incidence rates at national level.

In the study period, 47,087 (23.8%) had a laboratory confirmed SARS-CoV-2 result, of which 2,704 (5.7%) tested positive by LFD only and the rest were positive by PCR. Given the timing of vaccination rollout, the majority of positive tests that occurred in December 2020 were among residents prior to their first dose of vaccine (Figure 1).

In the analysis of COVID-19-related death, 196,924 individuals without a previous positive test prior to 8 December 2020 were included among which 10,608 (5.4%) died within 28 days of positive test, 3,935 (2.0%) died >28 days after a positive test, 24,260 (12.3%) died without a positive test and the remaining 158,121 (80.3%) did not die during the study period. The distribution of the time of COVID-19-related deaths is shown in Figure 1.

For the outcome of infection and death, aHRs for the time periods following first and second dose by any vaccine and vaccine are shown in Tables 2 and 3. Protection against infection and death was highest at 11–15 weeks and 1–4 weeks following second dose for BNT162b2 and ChAdOx-1, respectively.

**Table 2:** Adjusted HRs for infection by vaccination status for LTCF residents, England

**Vaccination status**	**Time since dose**	**Any**			**ChAdOx-1**			**BNT162b2**		
		**Person-time in days (unique individuals)** ^**a**^	**Events**	**Adjusted HR** ^**b**^	**Person-time in days (unique individuals)** ^**a**^	**Events**	**Adjusted HR** ^**b**^	**Person-time in days (unique individuals)** ^**a**^	**Events**	**Adjusted HR** ^**b**^
**Unvaccinated**		6,958,732 (190,202)	26,765		6,958,732 (190,202)	26,765		6,958,732 (190,202)	26,765	
**First dose**	1–2 wks	2,070,258 (153,383)	8,190	0.68 (0.62–0.74)	1,427,012 (105,580)	5,256	0.67 (0.6–0.75)	643,246 (47,803)	2,934	0.68 (0.6–0.78)
	3 wks	990,274 (143,432)	2,762	0.64 (0.57–0.73)	684,527 (99,045)	1,731	0.73 (0.63–0.86)	305,747 (44,387)	1,031	0.56 (0.48–0.67)
	4 wks	965,091 (139,327)	1,554	0.5 (0.43–0.59)	671,379 (96,744)	921	0.58 (0.48–0.7)	293,712 (42,583)	633	0.48 (0.39–0.59)
	5 wks	948,533 (136,661)	1,057	0.47 (0.4–0.56)	660,612 (95,140)	654	0.59 (0.47–0.73)	287,921 (41,521)	403	0.44 (0.36–0.55)
	6–7 wks	185,2109 (134,595)	1,190	0.46 (0.38–0.56)	129,0208 (93,718)	642	0.5 (0.4–0.62)	561,901 (40,877)	548	0.52 (0.41–0.66)
	8–10 wks	2,472,998 (130,173)	815	0.64 (0.5–0.82)	1,715,549 (90,634)	347	0.51 (0.38–0.68)	757,449 (39,539)	468	0.79 (0.59–1.06)
	11+ wks	1,112,436 (86,502)	254	0.83 (0.62–1.11)	768,455 (57,784)	181	0.94 (0.67–1.33)	343,981 (28,718)	73	0.63 (0.44–0.9)
**Second dose**	1–4 wks	3,432,288 (124,173)	239	0.4 (0.29–0.55)	2,401,640 (86,845)	119	0.39 (0.26–0.6)	1,030,648 (37,328)	120	0.38 (0.27–0.54)
	5–10 wks	5,037,822 (122,400)	179	0.47 (0.34–0.64)	3,521,278 (85,615)	134	0.54 (0.37–0.78)	1,516,544 (36,785)	45	0.34 (0.21–0.55)
	11–15 wks	4,035,312 (117,409)	384	0.45 (0.34–0.59)	2,810,444 (81,979)	327	0.48 (0.36–0.64)	1,224,868 (35,430)	57	0.31 (0.2–0.48)
	16–20 wks	3,757,167 (111,858)	1384	0.66 (0.54–0.81)	2,599,430 (77,764)	1090	0.72 (0.58–0.9)	1,157,737 (34,094)	294	0.55 (0.39–0.78)
	21+ wks	3,381,529 (99,696)	2,104	0.6 (0.49–0.74)	2,070,748 (68,221)	1,474	0.71 (0.57–0.9)	1,310,781 (31,475)	630	0.53 (0.42–0.68)

^a^Number of unique individuals at risk for any duration of time within each time period.

^b^Adjusted for gender, age group, case rate in local authority and deprivation, along with a cluster term for care home postcode. See Supplementary Figure S4, Supplementary Tables S1 and S2 in Supplementary data.

**Table 3:** Adjusted HRs for COVID-related death by vaccination status among LTCF residents, England

**Vaccination status**	**Time since dose**	**Any**			**ChAdOx-1**			**BNT162b2**		
		**Person-time in days (unique individuals)** ^**a**^	**Events**	**Adjusted HR** ^**b**^	**Person-time in days (unique individuals)** ^**a**^	**Events**	**Adjusted HR** ^**b**^	**Person-time in days (unique individuals)** ^**a**^	**Events**	**Adjusted HR** ^**b**^
**Unvaccinated**		6,931,978 (190,109)	7,425		6,931,978 (190,109)	7,425		6,931,978 (190,109)	7,425	
**First dose**	1–2 wks	2,070,228 (153,379)	2,125	0.59 (0.52–0.66)	1,426,998 (105,578)	1,364	0.58 (0.5–0.66)	643,230 (47,801)	761	0.6 (0.51–0.7)
	3–4 wks	1,955,365 (143,880)	812	0.41 (0.35–0.48)	1,355,906 (99,324)	485	0.49 (0.4–0.61)	599,459 (44,556)	327	0.35 (0.29–0.43)
	5–8 wks	3,697,628 (137,419)	347	0.33 (0.26–0.41)	2,575,162 (95,636)	178	0.37 (0.27–0.5)	1,122,466 (41,783)	169	0.34 (0.26–0.45)
	9+ wks	2,668,668 (124,523)	71	0.44 (0.3–0.63)	1,844,561 (86,556)	36	0.43 (0.26–0.71)	824,107 (37,967)	35	0.5 (0.32–0.78)
**Second dose**	1–4 wks	343,2248 (124,168)	18	0.15 (0.07–0.3)	240,1617 (86,843)	9	0.17 (0.06–0.42)	1,030,631 (37,325)	9	0.14 (0.06–0.33)
	5–10 wks	5,037,675 (122394)	15	0.19 (0.09–0.41)	3,521,162 (85,610)	10	0.18 (0.07–0.47)	1,516,513 (36,784)	5	0.19 (0.05–0.7)
	11–15 wks	4,035,106 (117,399)	43	0.21 (0.13–0.34)	2,810,271 (81,971)	39	0.22 (0.13–0.38)	1,224,835 (35,428)	4	0.09 (0.03–0.25)
	16–20 wks	3,756,005 (111,804)	193	0.35 (0.24–0.52)	2,598,423 (77,717)	155	0.39 (0.26–0.58)	1,157,582 (34,087)	38	0.27 (0.16–0.46)
	21+ wks	3,146,624 (94,716)	280	0.37 (0.25–0.53)	1,916,253 (64,662)	196	0.44 (0.3–0.67)	1,230,371 (30,054)	84	0.31 (0.2–0.49)

^a^Number of unique individuals at risk for any duration of time within each time period.

^b^Adjusted for gender, age group, case rate in local authority and deprivation, along with a cluster term for care home postcode. See Supplementary Figure S5, Supplementary Tables S3 and S4 in Supplementary data.

In post hoc analysis, there was evidence of waning of protection against infection after 16 weeks from second dose compared with the time period with best period of protection for both vaccines (Table 4). The estimates for waning of protection against death were limited by low precision.

**Table 4:** Post hoc comparison of adjusted HRs for dose 2 time periods, LTCF residents, England

Outcome	Vaccine type	Time period	Reference category^a^	Adjusted HR	*P* value
Infection	BNT162b2	16+ wks	11–15 wks	1.79 (1.15–2.78)	0.01
Infection	ChAdOx-1	16+ wks	1–4 wks	1.84 (1.14–2.96)	0.01
Death	BNT162b2	16+ wks	11–15 wks	3.36 (1.16–9.8)	0.03
Death	ChAdOx-1	16+ wks	1–4 wks	2.56 (0.95–6.92)	0.06

^a^Reference category indicates the time period following second dose when aHR was lowest for each vaccine.

In relation to the effect of dosing interval, we found that each additional week between first and second dose of ChAdOx-1 increased the risk of infection by 7% (95%CI 1–12%) in the first 4 weeks after second dose and had little effect thereafter (Supplementary Table S5). For BNT162b2, the corresponding estimates were 10% (4–16%) in the first 4 weeks and 9% (2–16%) after 4 weeks of the second dose. Of note, dosing interval did not have a detectable adverse effect against the outcome of death for either vaccine (Supplementary Table S6).

Supplementary Table S7 shows the aHRs for those with a previous positive test >90 days prior to 8 December 2020. In the subgroup analyses that included a main effects term for type of LTCF (nursing or residential), those in residential home had 10% (3–17%) increased hazard for infection and no increased hazard for death (1%, 95% CI −8% to 9%) compared with those resident in nursing homes. The estimates for models with an interaction term for time variables and residence type against infection and death are shown in Supplementary Figures S6 and S7.

## Discussion

Here we report real-world data on the effectiveness of one and two doses of the ChAdOx-1 and BNT162b2 vaccines against infection and death in residents of LTCFs. We show a modest protective effect of the first dose against infection that increases after second dose, and strong protective effect against COVID-19-related death, particularly after receipt of second dose.

We estimated that relative to unvaccinated individuals, VE for infection was highest for ChAdOx-1 at 61% (40–74%) at 1–4 weeks and for BNT162b2 at 69% (52–80%) at 11–15 weeks following the second dose. Against death, VE was highest for ChAdOx-1 at 83% (58–94%) at 1–4 weeks and for BNT162b2 at 91% (75–97%) at 11–15 weeks following second dose. Although our findings are consistent with the estimates reported by the VIVALDI team, we present data for a longer follow-up period after second dose [11]. Considering the CIs for VE by vaccine type across all time periods, the vaccines were broadly comparable in terms of protection offered against infection and death.

We were able to estimate VE against infection regardless of presence of symptoms due to the implementation of regular testing programme for LTCFs in England. Due to clustering of highly vulnerable individuals and frequent contact with staff providing care in the LTCF, their risk is elevated compared with older individuals living in the wider community [12]. As such, the VE estimates will inevitably be lower than that reported in a test-negative design, which relies on individuals who access testing in the presence of symptoms [13, 14]. Test positivity in LTCF residents, regardless of symptoms, has implications for individual care and infection control within LTCFs. Given that there are other studies investigating VE against symptomatic infection, this study was designed specifically to estimate VE against infection regardless of symptoms in a highly vulnerable population resident in LTCFs with access to a regular SARS-CoV-2 testing programme.

We found that protection against death was highest after the first dose at 5–8 weeks for BNT162b2 and ChAdOx-1 with VE estimated at 66% (55–74%) and 63% (50–73%), respectively. Given that VE estimates for death are over 60% at 8 weeks for either, the UK policy of maximising first dose vaccine uptake amongst the most vulnerable by increasing the interval to second dose in light of high incidence and vaccine supply constraints is likely to have reduced overall mortality. Following the second dose, VE was highest at 11–15 weeks for BNT162b2 at 91% (75–97%) and for ChAdOx-1 at 83% (58–94%) at 1–4 weeks. This is in keeping with other real-world data [6, 7, 15].

In this study, we found that for each additional week in the interval between first and second dose, the risk of infection in the first 4 weeks following the second dose increased marginally and was similar for ChAdOx-1 and BNT162b2. However, the increased risk of infection persisted for BNT162b2 beyond 4 weeks by 9% (2–16%) for each week but not for ChAdOx-1. This may be in part due to the fact that the manufacturer recommended dosing interval for ChAdOx-1 is 8–12 weeks and for BNT162b2 is 3–6 weeks. The dosing interval for BNT162b2 used in the UK is different to some other countries and as such our findings for this vaccine may not generalise to other settings. Reassuringly, we found no evidence that dosing interval had any adverse effect on the more significant of COVID-related death for either vaccine.

The start of the study period coincided with the emergence of the Alpha (B.1.17) variant, which remained dominant until mid-May 2021 [16]. However, by the end of the study period on 30 September 2021, the Delta variant accounted for ~99% of sequenced and 97% genotyped cases [17]. We were unable to estimate the effect of vaccines by variant type in LTCF residents due to few residents reaching the endpoint of infection or death after the second dose. Other studies providing variant-specific VE have been published [15, 18].

This study has several strengths. First, VE analysis was conducted for all persons over 65 years of age living in LTCFs in England, who are tested regularly irrespective of symptoms, using comprehensive data linking SARS-CoV-2 test results, immunisation and mortality records. Second, VE was estimated in a time-varying regression model that adjusted for both the time following vaccination and calendar time (through the baseline hazard) and weekly incidence rate in the local authority to effectively adjust for background risk of exposure at a more granular level. Deprivation was included in the model as it is known to influence both risk of exposure as well as vaccine hesitancy and uptake. Third, the size of the dataset allowed evaluation of the effect of dosing interval on infection and COVID-related death in this population. Fourth, we were able to assess VE based on a large cohort of LTCF residents over a longer period than most other published studies.

There are several limitations to this study. First, we were unable to adjust for comorbidities at the individual level as data were not available. Second, data on cycle threshold values for positive samples, clinical data, or vaccination uptake rates for staff were not available for linkage and therefore could not be accounted for in the VE estimates. Third, we note that our analyses were subject to the competing risk of death from other causes, though we consider it unlikely that that vaccination might influence death from other causes in this older population. Finally, the VE estimates presented in this paper are not variant-specific. Despite the limitation, this study provides valuable data on real-world effectiveness of vaccines in this vulnerable cohort against important outcome measures.

## Conclusions

Compared with unvaccinated residents, vaccination with one dose of BNT162b2 or ChAdOx-1 provided moderate protection against infection and death in residents of LTCFs. Protection against death improved after two doses. However, some waning of protection over time was noted. Ongoing surveillance on possible waning of protection against infection and severe outcome is warranted.

## Supplementary Material

aa-21-1873-File002_afac115
