# Supplementary material for: Effectiveness of BNT162b2 and ChAdOx-1 vaccines in residents of long-term care facilities in England using a time-varying proportional hazards model
Source: Age Ageing. 2022 May 20;51(5):afac115. doi: 10.1093/ageing/afac115 (PMC9382883; doi:10.1093/ageing/afac115)

Supplementary data

COVID vaccine effectiveness in LTCF residents, 2020-21, England

This appendix has been provided by the authors to give readers additional information about the work.

Table of contents

[1. Study methods 3](#_Toc97543772)

[1.1. Design, period and setting 3](#_Toc97543773)

[1.2. Exclusions 3](#_Toc97543774)

[1.3. Data sources 3](#_Toc97543775)

[1.4. Covariates 3](#_Toc97543776)

[1.5. Definitions 4](#_Toc97543777)

[1.6. Missing data 5](#_Toc97543778)

[1.7. Time varying Cox regression 5](#_Toc97543779)

[1.8. Post-hoc/additional analyses 6](#_Toc97543780)

List of figures

[**Figure S1**. Distribution of interval between first and second dose among LTCF residents 7](#_Toc97544202)

[**Figure S2**. Follow-up period at each time period for individuals in study 8](#_Toc97544203)

[**Figure S3**. Rolling 7-day SARS CoV-2 incidence rate, England 9](#_Toc97544204)

[**Figure S4**. Adjusted hazard ratios for infection 10](#_Toc97544205)

[**Figure S5**. Adjusted hazard ratios for COVID-related death 11](#_Toc97544206)

[**Figure S6**. Adjusted hazard ratios for infection (residence type) 26](#_Toc97544207)

[**Figure S7**. Adjusted hazard ratios for death (residence type) 27](#_Toc97544208)

List of tables

[**Table S1**. Illustration of case data in time varying Cox regression 6](#_Toc97544209)

[**Table S2**. Adjusted hazard ratios for infection (any vaccine) 12](#_Toc97544210)

[**Table S3**. Adjusted hazard ratios for infection (vaccine type) 14](#_Toc97544211)

[**Table S4**. Adjusted hazard ratios for death (any vaccine) 16](#_Toc97544212)

[**Table S5**. Adjusted hazard ratios for death (vaccine type) 18](#_Toc97544213)

[**Table S6**. Adjusted hazard ratios for infection (dosing interval) 20](#_Toc97544214)

[**Table S7**. Adjusted hazard ratios for death (dosing interval) 22](#_Toc97544215)

[**Table S8**. Adjusted hazard ratios for infection (those with previous positive test) 24](#_Toc97544216)

# Study methods

## Design, period and setting

In this observational population study, we analysed surveillance data from the study period 8 December 2020 to 30 September 2021.
The study population were residents greater than 65 years in LTCFs in England with at least two recorded tests for SARS CoV-2 and at least one test during the study period. The care home flag is generated from the NHS England and Improvement Master Patient Index and uses a Unique Property Reference Numbers and NHS-addresses linked to care home Care Quality Commission addresses. An Individual is given this flag if they have ever lived in a care home since the beginning of the vaccine programme and is updated monthly.

## Exclusions

- Individuals records as staff working at LTCF as reported in data collected at the time of testing
- Individuals who were resident in more than one LTCF during the study period, based on postcode data obtained at the time of their regular testing
- Residents with a positive result prior to 8 December 2020 (excluded from primary analysis)
- Individuals with heterologous vaccines were retained from start till the date of receipt of second dose but excluded for all periods after second dose
- Individuals with missing core identifiers for data linkage

## Data sources

- Immunisation data - Individual vaccination records in national immunisation management system (NIMS) database, a comprehensive database of all COVID-19 immunisations in England, were linked to testing data using NHS number, date of birth, first name and surname and postcode.
- Testing data - Data on all test results (negative and positive) from lateral flow device (LFD) and polymerase chain reaction (PCR) testing between 8 December 2020 and 30 September 2021 were extracted. Negative tests up to 21 days prior to a positive result were removed from the dataset as these could indicate false negative results. For anyone with both a positive LFD and a positive PCR, only the PCR result was retained.
- ONS data - Data on all cause death and date of death for all individuals in the study was sourced from the Office for National Statistics (ONS).
- Prevalence data - Weekly SARS-CoV-2 incidence rate per 100,000 population were calculated at the Local Authority level and linked to individuals based on postcode.

## Covariates

- Age group – we grouped age in five-year bands from 65 to 90 years (65-69, 70-74, 75-79, 80-84, 85-89) and an open category for those aged 90 years and above (90+)
- Sex – with female as reference category
- Case rate - As prevalence of infection in the communities where the individuals are resident varied during the duration of study period, we used 7-day moving average case rate (100 cases per 100,000 population) at the lower tier local authority level calculated for each day of the study period as a covariate. There are ~330 LTLAs (local authority districts, unitary authorities, metropolitan districts, London boroughs) in England with an average population of 170,000 residents. Case rate at LTLA level is good indicator for level of prevalence in the community and hence exposure risk during the time periods individuals contributed to the study.
- Index of deprivation - The index of multiple deprivation is a national indicator of level of deprivation on the basis of small geographic areas of residence. It is a combined measure of deprivation based on a total of 37 separate indicators that have been grouped into seven domains according to respective weights (<https://www.gov.uk/government/statistics/english-indices-of-deprivation-2019>). We combined IMD deciles to form quintiles, with 1 being least deprived and 5 being most deprived.
- Care home postcode (random cluster term in models) – the postcode of the individual based on testing and NIMS data We originally intended to included ethnicity as a covariate. However, we found that the vast majority of LTCF residents in the dataset were of White ethnicity and we had limited confidence that the ethnicity data was reliable for this cohort. Other VE analyses undertaken by PHE indicated that ethnicity was not an important covariate for VE.

We originally intended to include ethnicity as a covariate. However, we found that the vast majority of LTCF residents in the dataset were of White ethnicity and we had limited confidence that the ethnicity data was reliable for this cohort. Other VE analyses undertaken by PHE indicated that ethnicity was not an important covariate for VE.

## Definitions

- Infection with SARS-CoV-2: confirmed infection by positive RNA detection (PCR test) or lateral flow device (LFD) testing
- COVID-related death: all-cause death within 28 days of confirmed infection with SARS-CoV-2
- Date first at risk for dose 1: study start date (8 December 2020) if individual had a recorded test in the six weeks prior to 8 Dec 2020 or date of first test if testing occurred before receipt of second dose. Not at risk if date of first test after receipt of second dose.
- Date of risk end for dose 1: earliest of date of positive test, date of last test in study period or date of receipt of second dose of vaccine
- Date first at risk for dose 2: date of receipt of vaccine dose 2 if tested prior or date of first test if tested after dose 2 receipt
- Date of risk end for dose 2: earliest of date of positive test, date of last test in study period or receipt of third dose of vaccine
- Time intervals: to assess VE for any vaccine
  - Infection outcome: 1-2, 3, 4, 5, 6-7, 8-10 and 11+ weeks for first dose and 1-4, 5-10, 11-15, 16-20, 21+ weeks for second dose
  - Death outcome: 1-2, 3-4, 4-8, 9+ weeks for first dose and 1-4, 5-10, 11-15, 16-20, 21+ weeks for second dose
- Vaccination status: the above time interval variables were further classified by vaccine type (to assess VE by vaccine type).

## Missing data

The overall proportion of individuals with missing data on key covariates was very low (<0.01%). Hence, they were retained in the study dataset and included in descriptive analyses. These individuals would have been automatically dropped if missing relevant data in the Cox regression models.

## Time varying Cox regression

A fundamental assumption of Cox proportional hazards model is that factors under study (covariates) either increase or decrease the baseline hazard function with an effect that is constant over time. Time-varying Cox regression is a technique that can be used to model changing effects of covariates over time, thus relaxing the requirement of proportional hazards. In the current study, individuals were vaccinated with the first or second dose of vaccine over a period of several months, hence their risk of getting infected varies according to their dates of vaccination.

The primary covariate in the study is the time period for vaccine effectiveness (VE) estimation which in addition to unvaccinated has 12 vaccination categories: 1-2, 3, 4, 5, 6-7, 8-10 and 11+ weeks for first dose and 1-4, 5-10, 11-15, 16-20, 21+ weeks for second dose. VE for all of the above time periods are estimated in comparison to unvaccinated individuals. To incorporate the time period as a time varying covariate, each study participant’s follow-up time was calculated as the number of days beginning 8 December 2020 (day 1, the study start date) so 9 December 2020 is day 2 and so on. To illustrate further, the following example may be helpful, where the event of interest is infection.

- Individual A enters study on study on Day 1 (8 Dec 2020), gets infected on day 8 (15 Dec 2020) and their follow-up therefore ends at this point.
- Individual B enters study on Day 1 (8 Dec 2020), receives first dose of vaccine on day 12 (20 Dec 2020) and gets infected with Covid on day 44 (20 Jan 2021). Hence the individual contributes 11 days at-risk to the unvaccinated time period, then is at risk from days 12 to 44 over four different periods following dose 1 and follow-up stops at day 44 when they get infected. As they developed infection prior to second dose, they do not contribute to the time periods relating to second dose.
- Individual C enters study on day 25 (1 Jan 2021), gets first dose of vaccine on day 56 (1 Feb 2021), receives second dose of vaccine on day 145 (1 May 2021) and gets infected on day 186 (11 June 2021) and follow-up stops at this point.
- Individual D enters study on receiving first dose vaccine on day 60 (5 Feb 2021), receives second dose on day 84 (1 Mar 2021) and gets infected on day 93 (10 Mar 2021).

Each individual’s follow-up data is split to specified time intervals, with one row of data for each interval. as shown in Table 1 below. The time period is a categorical time-varying covariate and over time individuals ‘move on’ to further categories as they receive vaccine doses and time elapses from these doses. For each row of time variable per individual, event coding is a binary variable (0 if no event, 1 if event occurred). Including VE period as a time-varying covariate in a Cox regression model permits the estimation of hazards ratios (which is then converted to VE) for vaccine doses that vary over time for individuals entering the study.

**Table S1**. Illustration of case data in time varying Cox regression

| **Vaccination** | **VE time periods** | **Individual A** | **Individual B** | **Individual C** | **Individual D** |
| --- | --- | --- | --- | --- | --- |
| **Unvaccinated** | Unvaccinated | 1-8 | 1-11 | 25-55 |  |
| **First dose** | 1-2 weeks |  | 12-26 | 56-69 | 60-73 |
| **First dose** | 3 weeks |  | 27-33 | 70-76 | 74-80 |
| **First dose** | 4 weeks |  | 34-40 | 76-82 | 81-84 |
| **First dose** | 5 weeks |  | 41-44 | 83-89 |  |
| **First dose** | 6-7 weeks |  |  | 90-103 |  |
| **First dose** | 8-10 weeks |  |  | 104-124 |  |
| **First dose** | 11+ weeks |  |  | 124-145 |  |
| **Second dose** | 1-4 weeks |  |  | 145-173 | 84-93 |
| **Second dose** | 5-10 weeks |  |  | 174-185 |  |

## Post-hoc/additional analyses

- Waning of protection: Given the growing literature of waning of protection associated with Covid vaccines, we refitted models to assess waning against infection and death for second dose time variables. For this, we chose the time period with lowest aHR for each vaccine type as reference category and refitted models to estimate the effect of the final time period (16+ weeks).
- Effect of dosing interval: We created a variable for interval between receipt of first and second dose in complete weeks. We subtracted this dosing interval variable by 10 weeks (median interval for all individuals in study dataset) to interpret the VE as relevant to a ‘typical’ individual. We hypothesised that the effect of dosing interval might have different effects in the immediate period (1-4 weeks) and later period after second dose, because the former would include ongoing effects of the first dose, and included separate terms for interval for these periods. Further classifying by vaccine type, four terms were created - scaled interval for each of BNT162b2 and ChAdOx-1 for the first four weeks and >4 weeks after second dose. We ran two models with these four additional covariates against infection and death.
- VE among those with previous positive test: We ran a model to assess VE against infection for those who had had a previous positive test at least 90 days prior to study start date.
- Subgroup analysis for residential and nursing homes: We linked our core dataset to the registry of care homes held by Care Quality Commission. This registry contains details of whether each care home in England provided nursing care, residential care or both. After excluding homes providing both types of care, we modelled the effect of care home type (residential vs nursing) as main effects and interaction against the outcome of infection and death outcome.

**Figure S****1**. Distribution of interval between first and second dose among LTCF residents


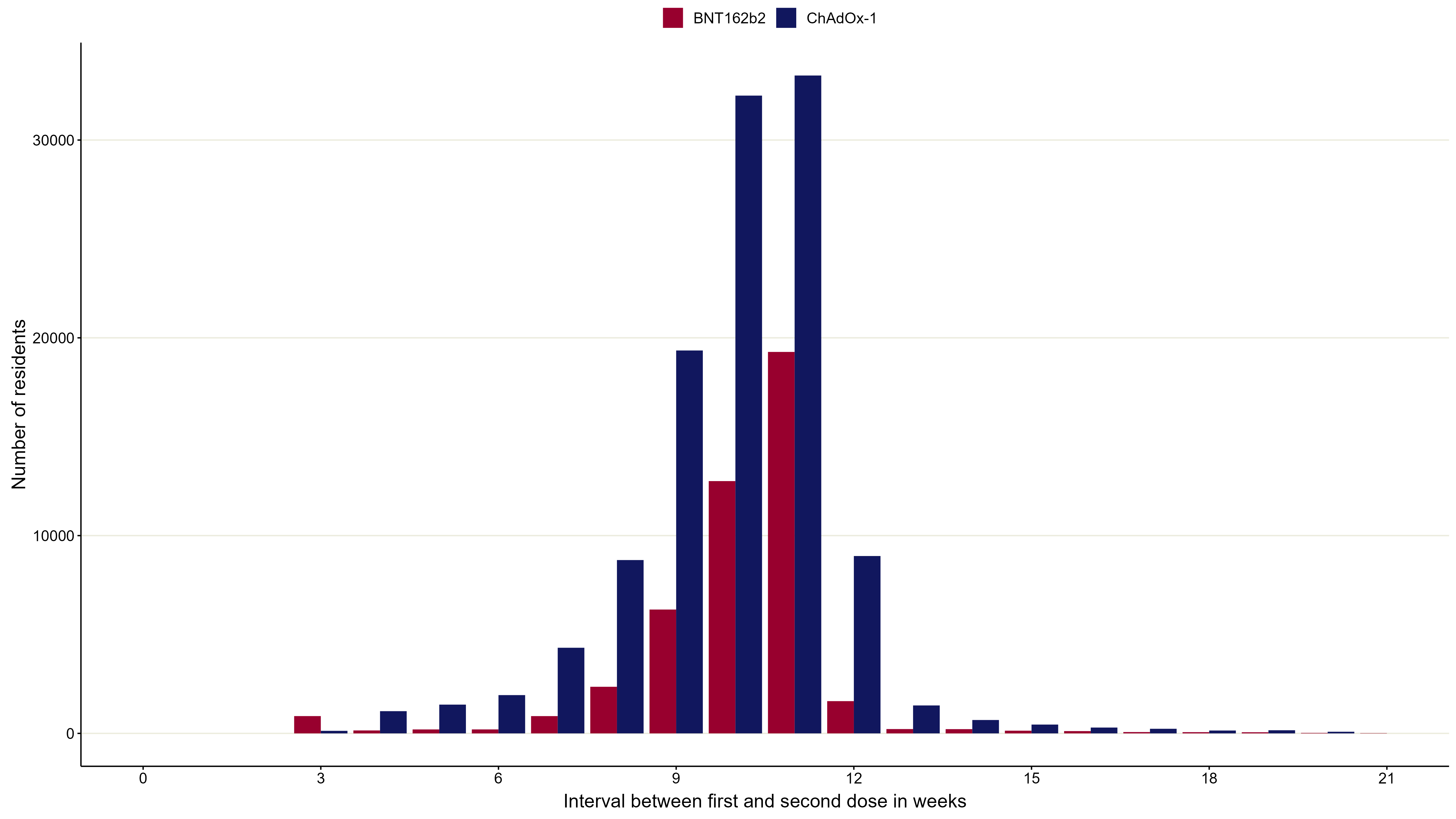


**Figure S****2**. Follow-up period at each time period for individuals in study


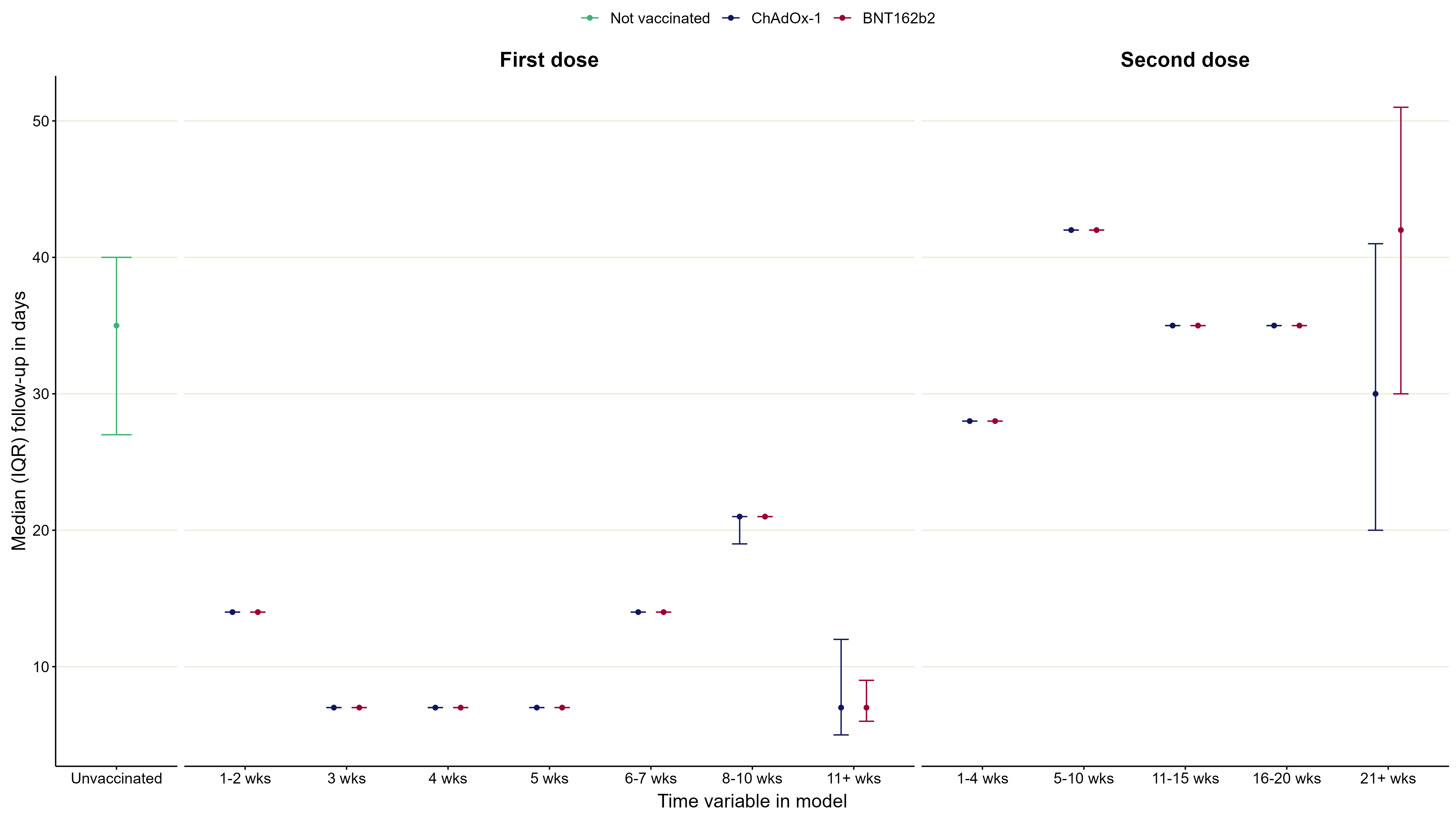


**Figure S****3**. Rolling 7-day SARS CoV-2 incidence rate, England


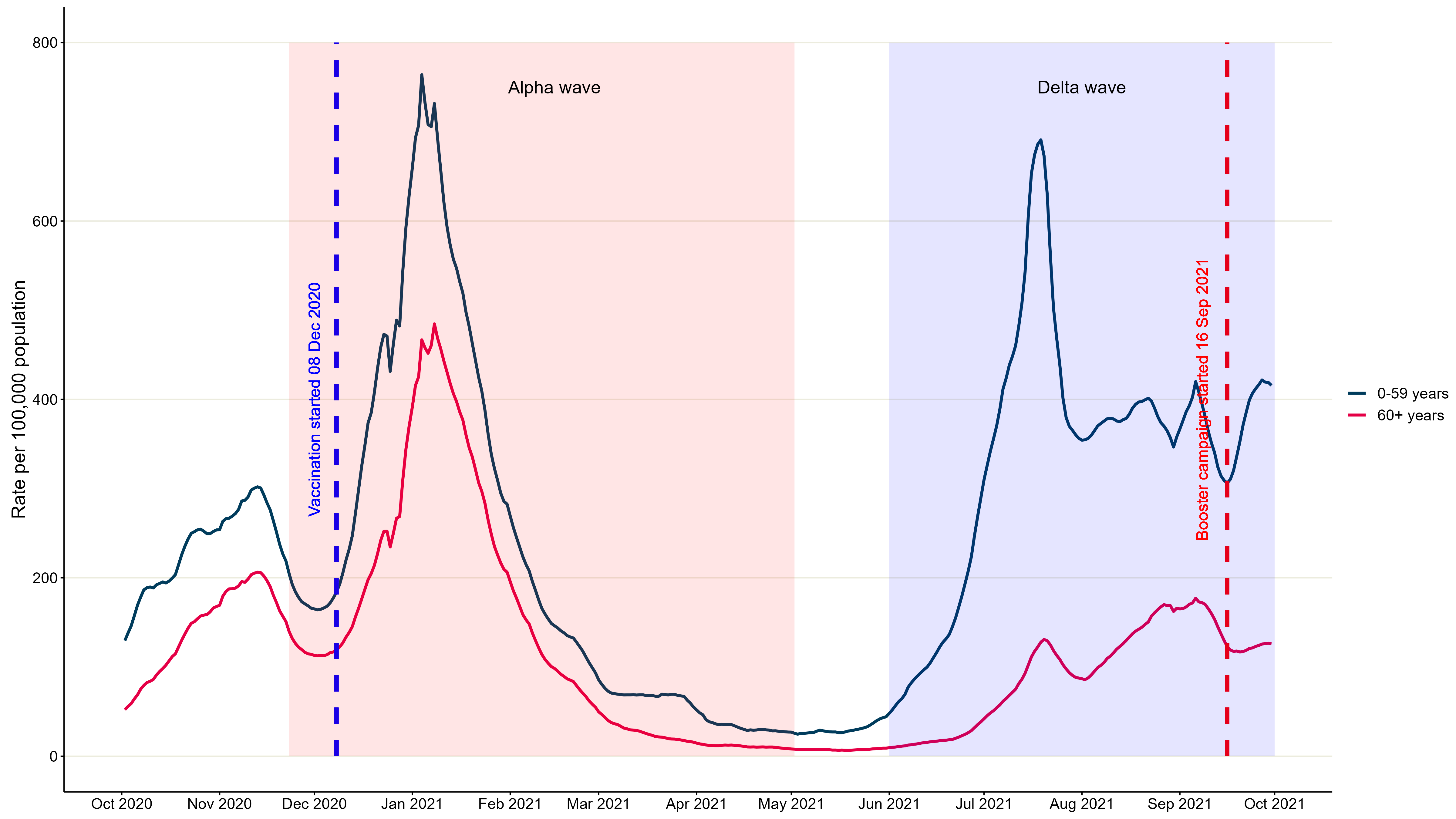


**Figure S****4**. Adjusted hazard ratios for infection


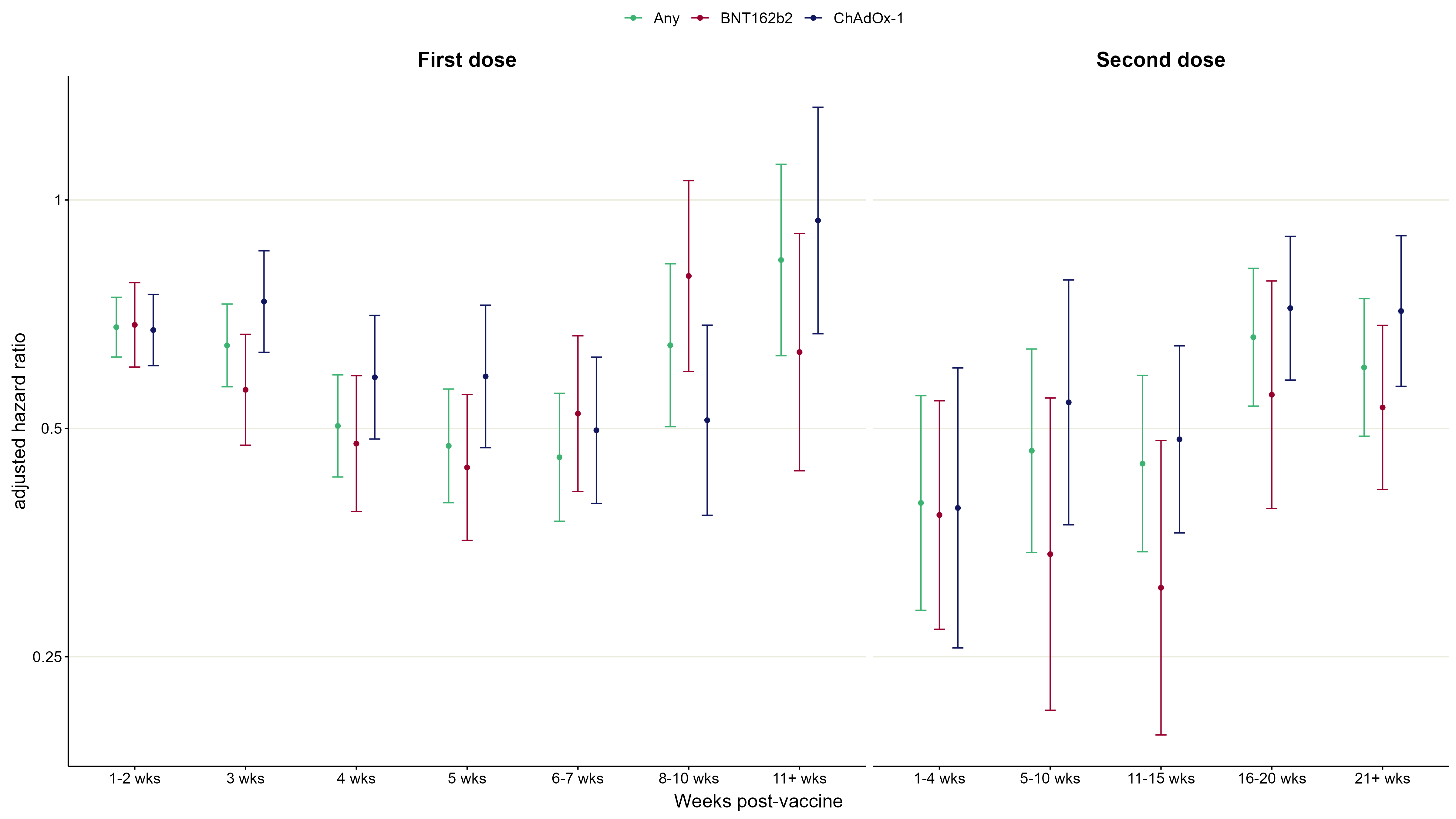


**Figure S****5**. Adjusted hazard ratios for COVID-related death


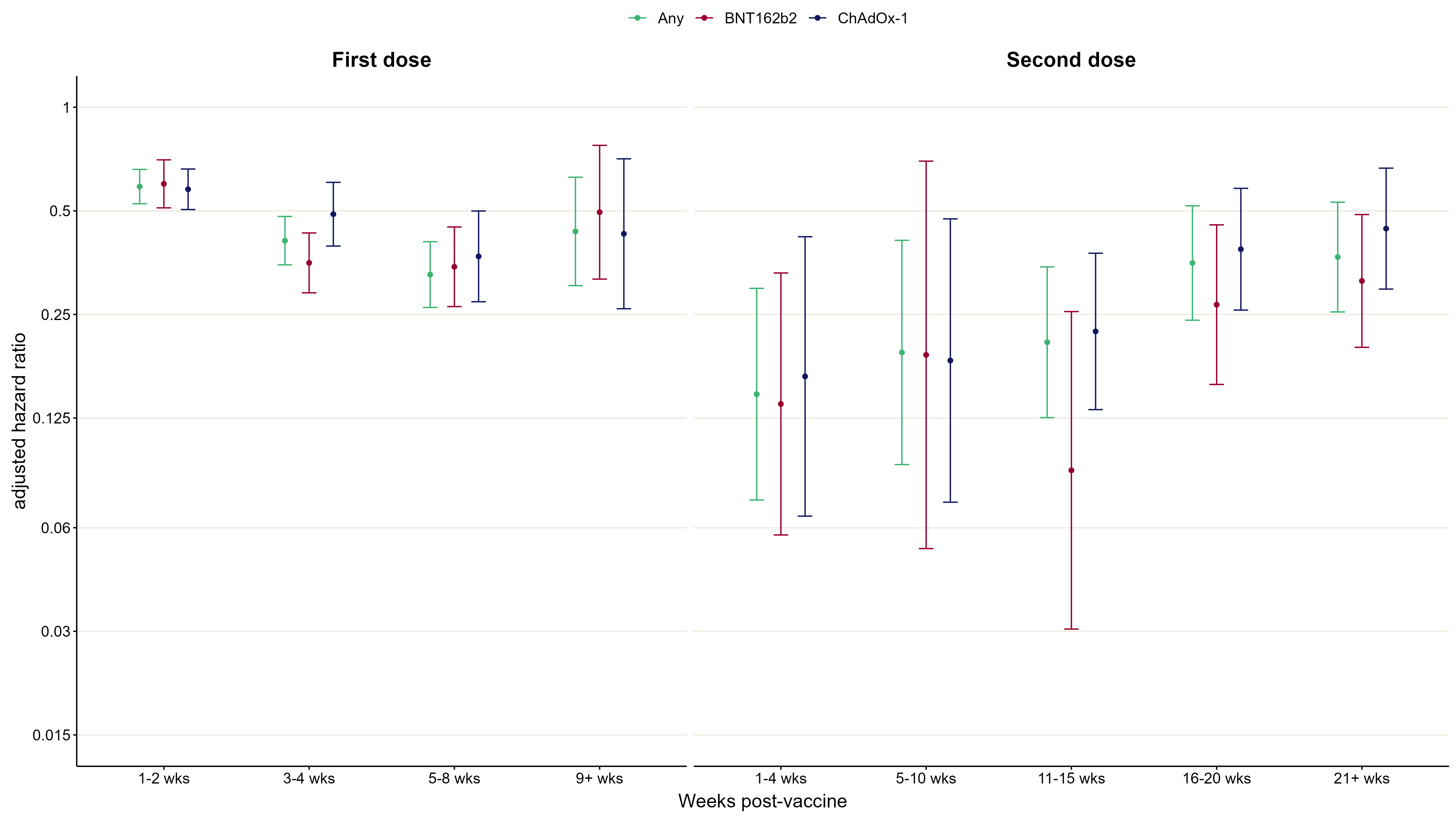


**Table S2**. Adjusted hazard ratios for infection (any vaccine)

| **Variable** | **Levels** | **Number of individuals** | **Number of events** | **Adjusted hazard ratio** |
| --- | --- | --- | --- | --- |
| **Unvaccinated** |  | 190202 | 26765 | Reference |
| **First dose** | 1-2 wks | 153383 | 8190 | 0.68 (0.62-0.74) |
|  | 3 wks | 143432 | 2762 | 0.64 (0.57-0.73) |
|  | 4 wks | 139327 | 1554 | 0.5 (0.43-0.59) |
|  | 5 wks | 136661 | 1057 | 0.47 (0.4-0.56) |
|  | 6-7 wks | 134595 | 1190 | 0.46 (0.38-0.56) |
|  | 8-10 wks | 130173 | 815 | 0.64 (0.5-0.82) |
|  | 11+ wks | 86502 | 254 | 0.83 (0.62-1.11) |
| **Second dose** | 1-4 wks | 124173 | 239 | 0.4 (0.29-0.55) |
|  | 5-10 wks | 122400 | 179 | 0.47 (0.34-0.64) |
|  | 11-15 wks | 117409 | 384 | 0.45 (0.34-0.59) |
|  | 16-20 wks | 111858 | 1384 | 0.66 (0.54-0.81) |
|  | 21+ wks | 99696 | 2104 | 0.6 (0.49-0.74) |
| **Age group** | 65-69 years | 9346 | 1871 | Reference |
|  | 70-74 years | 16303 | 3619 | 1.15 (1.09-1.22) |
|  | 75-79 years | 23977 | 5626 | 1.25 (1.19-1.33) |
|  | 80-84 years | 37221 | 9247 | 1.36 (1.29-1.44) |
|  | 85-89 years | 48836 | 11999 | 1.36 (1.29-1.44) |
|  | 90+ years | 61450 | 14515 | 1.32 (1.25-1.4) |
| **Case rate** |  |  |  | 1.07 (1.05-1.08) |
| **Sex** | Female | 138662 | 32382 | Reference |
|  | Male | 58471 | 14495 | 1.14 (1.11-1.16) |
| **Relative deprivation** | 1 (least deprived) | 34669 | 8006 | Reference |
|  | 2 | 38828 | 9169 | 1.02 (0.93-1.12) |
|  | 3 | 41873 | 10118 | 1.07 (0.97-1.17) |
|  | 4 | 43117 | 9920 | 1.01 (0.92-1.11) |
|  | 5 (most deprived) | 38646 | 9664 | 1.13 (1.02-1.25) |

**Table S3**. Adjusted hazard ratios for infection (vaccine type)

| **Variable** | **Levels** | **Vaccine type** | **Number of individuals** | **Number of events** | **Adjusted hazard ratio** |
| --- | --- | --- | --- | --- | --- |
| **Unvaccinated** |  |  | 190202 | 26765 | Reference |
| **First dose** | 1-2 wks | ChAdOx-1 | 105580 | 5256 | 0.67 (0.6-0.75) |
|  |  | BNT162b2 | 47803 | 2934 | 0.68 (0.6-0.78) |
|  | 3 wks | ChAdOx-1 | 99045 | 1731 | 0.73 (0.63-0.86) |
|  |  | BNT162b2 | 44387 | 1031 | 0.56 (0.48-0.67) |
|  | 4 wks | ChAdOx-1 | 96744 | 921 | 0.58 (0.48-0.7) |
|  |  | BNT162b2 | 42583 | 633 | 0.48 (0.39-0.59) |
|  | 5 wks | ChAdOx-1 | 95140 | 654 | 0.59 (0.47-0.73) |
|  |  | BNT162b2 | 41521 | 403 | 0.44 (0.36-0.55) |
|  | 6-7 wks | ChAdOx-1 | 93718 | 642 | 0.5 (0.4-0.62) |
|  |  | BNT162b2 | 40877 | 548 | 0.52 (0.41-0.66) |
|  | 8-10 wks | ChAdOx-1 | 90634 | 347 | 0.51 (0.38-0.68) |
|  |  | BNT162b2 | 39539 | 468 | 0.79 (0.59-1.06) |
|  | 11+ wks | ChAdOx-1 | 57784 | 181 | 0.94 (0.67-1.33) |
|  |  | BNT162b2 | 28718 | 73 | 0.63 (0.44-0.9) |
| **Second dose** | 1-4 wks | ChAdOx-1 | 86845 | 119 | 0.39 (0.26-0.6) |
|  |  | BNT162b2 | 37328 | 120 | 0.38 (0.27-0.54) |
|  | 5-10 wks | ChAdOx-1 | 85615 | 134 | 0.54 (0.37-0.78) |
|  |  | BNT162b2 | 36785 | 45 | 0.34 (0.21-0.55) |
|  | 11-15 wks | ChAdOx-1 | 81979 | 327 | 0.48 (0.36-0.64) |
|  |  | BNT162b2 | 35430 | 57 | 0.31 (0.2-0.48) |
|  | 16-20 wks | ChAdOx-1 | 77764 | 1090 | 0.72 (0.58-0.9) |
|  |  | BNT162b2 | 34094 | 294 | 0.55 (0.39-0.78) |
|  | 21+ wks | ChAdOx-1 | 68221 | 1474 | 0.71 (0.57-0.9) |
|  |  | BNT162b2 | 31475 | 630 | 0.53 (0.42-0.68) |
| **Age group** | 65-69 years |  | 9346 | 1871 | Reference |
|  | 70-74 years |  | 16303 | 3619 | 1.15 (1.09-1.22) |
|  | 75-79 years |  | 23977 | 5626 | 1.26 (1.19-1.33) |
|  | 80-84 years |  | 37221 | 9247 | 1.36 (1.29-1.44) |
|  | 85-89 years |  | 48836 | 11999 | 1.36 (1.29-1.44) |
|  | 90+ years |  | 61450 | 14515 | 1.32 (1.25-1.4) |
| **Case rate** |  |  |  |  | 1.07 (1.05-1.08) |
| **Sex** | Female |  | 138662 | 32382 | Reference |
|  | Male |  | 58471 | 14495 | 1.14 (1.11-1.16) |
| **Relative deprivation** | 1 (least deprived) |  | 34669 | 8006 | Reference |
|  | 2 |  | 38828 | 9169 | 1.02 (0.92-1.12) |
|  | 3 |  | 41873 | 10118 | 1.06 (0.97-1.17) |
|  | 4 |  | 43117 | 9920 | 1.01 (0.92-1.11) |
|  | 5 (most deprived) |  | 38646 | 9664 | 1.13 (1.02-1.25) |

**Table S4**. Adjusted hazard ratios for death (any vaccine)

| **Variable** | **Levels** | **Number of individuals** | **Number of events** | **Adjusted hazard ratio** |
| --- | --- | --- | --- | --- |
| **Unvaccinated** |  | 190109 | 7425 | Reference |
| **First dose** | 1-2 wks | 153379 | 2125 | 0.59 (0.52-0.66) |
|  | 3-4 wks | 143880 | 812 | 0.41 (0.35-0.48) |
|  | 5-8 wks | 137419 | 347 | 0.33 (0.26-0.41) |
|  | 9+ wks | 124523 | 71 | 0.44 (0.3-0.63) |
| **Second dose** | 1-4 wks | 124168 | 18 | 0.15 (0.07-0.3) |
|  | 5-10 wks | 122394 | 15 | 0.19 (0.09-0.41) |
|  | 11-15 wks | 117399 | 43 | 0.21 (0.13-0.34) |
|  | 16-20 wks | 111804 | 193 | 0.35 (0.24-0.52) |
|  | 21+ wks | 94716 | 280 | 0.37 (0.25-0.53) |
| **Age group** | 65-69 years | 9332 | 242 | Reference |
|  | 70-74 years | 16287 | 593 | 1.5 (1.29-1.74) |
|  | 75-79 years | 23950 | 1115 | 2.06 (1.79-2.38) |
|  | 80-84 years | 37206 | 2064 | 2.62 (2.28-3.01) |
|  | 85-89 years | 48813 | 3044 | 3.08 (2.69-3.54) |
|  | 90+ years | 61431 | 4271 | 3.59 (3.13-4.12) |
| **Case rate** |  |  |  | 1.07 (1.05-1.09) |
| **Sex** | Female | 138586 | 6890 | Reference |
|  | Male | 58433 | 4439 | 1.84 (1.77-1.92) |
| **Relative deprivation** | 1 (least deprived) | 34647 | 1749 | Reference |
|  | 2 | 38799 | 2215 | 1.1 (0.96-1.26) |
|  | 3 | 41849 | 2487 | 1.17 (1.02-1.33) |
|  | 4 | 43095 | 2441 | 1.1 (0.96-1.25) |
|  | 5 (most deprived) | 38629 | 2437 | 1.24 (1.08-1.42) |

**Table S5**. Adjusted hazard ratios for death (vaccine type)

| **Variable** | **Levels** | **Vaccine type** | **Number of individuals** | **Number of events** | **Adjusted hazard ratio** |
| --- | --- | --- | --- | --- | --- |
| **Unvaccinated** |  |  | 190202 | 26765 | Reference |
| **First dose** | 1-2 wks | ChAdOx-1 | 105580 | 5256 | 0.67 (0.6-0.75) |
|  |  | BNT162b2 | 47803 | 2934 | 0.68 (0.6-0.78) |
|  | 3 wks | ChAdOx-1 | 99045 | 1731 | 0.73 (0.63-0.85) |
|  |  | BNT162b2 | 44387 | 1031 | 0.56 (0.47-0.66) |
|  | 4 wks | ChAdOx-1 | 96744 | 921 | 0.58 (0.48-0.7) |
|  |  | BNT162b2 | 42583 | 633 | 0.48 (0.39-0.59) |
|  | 5 wks | ChAdOx-1 | 95140 | 654 | 0.59 (0.47-0.73) |
|  |  | BNT162b2 | 41521 | 403 | 0.44 (0.35-0.55) |
|  | 6-7 wks | ChAdOx-1 | 93718 | 642 | 0.5 (0.4-0.62) |
|  |  | BNT162b2 | 40877 | 548 | 0.52 (0.41-0.66) |
|  | 8-10 wks | ChAdOx-1 | 90634 | 347 | 0.53 (0.4-0.71) |
|  |  | BNT162b2 | 39539 | 468 | 0.8 (0.6-1.07) |
|  | 11+ wks | ChAdOx-1 | 57784 | 181 | 1.02 (0.73-1.43) |
|  |  | BNT162b2 | 28718 | 73 | 0.67 (0.46-0.96) |
| **Second dose** | 1-4 wks | ChAdOx-1 | 86845 | 119 | 0.44 (0.29-0.69) |
|  |  | BNT162b2 | 37328 | 120 | 0.49 (0.32-0.73) |
|  | 5-10 wks | ChAdOx-1 | 85615 | 134 | 0.61 (0.42-0.89) |
|  |  | BNT162b2 | 36785 | 45 | 0.43 (0.26-0.7) |
|  | 11-15 wks | ChAdOx-1 | 81979 | 327 | 0.52 (0.39-0.7) |
|  |  | BNT162b2 | 35430 | 57 | 0.32 (0.2-0.5) |
|  | 16-20 wks | ChAdOx-1 | 77764 | 1090 | 0.77 (0.62-0.96) |
|  |  | BNT162b2 | 34094 | 294 | 0.57 (0.41-0.81) |
|  | 21+ wks | ChAdOx-1 | 68221 | 1474 | 0.76 (0.6-0.95) |
|  |  | BNT162b2 | 31475 | 630 | 0.57 (0.44-0.74) |
| **Age group** | 65-69 years |  | 9346 | 1871 | Reference |
|  | 70-74 years |  | 16303 | 3619 | 1.15 (1.09-1.22) |
|  | 75-79 years |  | 23977 | 5626 | 1.26 (1.19-1.33) |
|  | 80-84 years |  | 37221 | 9247 | 1.36 (1.29-1.44) |
|  | 85-89 years |  | 48836 | 11999 | 1.36 (1.29-1.44) |
|  | 90+ years |  | 61450 | 14515 | 1.32 (1.25-1.4) |
| **Case rate** |  |  |  |  | 1.07 (1.05-1.08) |
| **Sex** | Female |  | 138662 | 32382 | Reference |
|  | Male |  | 58471 | 14495 | 1.14 (1.11-1.16) |
| **Relative deprivation** | 1 (least deprived) |  | 34669 | 8006 | Reference |
|  | 2 |  | 38828 | 9169 | 1.02 (0.93-1.12) |
|  | 3 |  | 41873 | 10118 | 1.06 (0.97-1.17) |
|  | 4 |  | 43117 | 9920 | 1.01 (0.92-1.11) |
|  | 5 (most deprived) |  | 38646 | 9664 | 1.13 (1.02-1.25) |
| **Scaled interval (weeks)** | AZ: 1-4 weeks |  |  |  | 1.07 (1.01-1.12) |
|  | PF: 1-4 weeks |  |  |  | 1.1 (1.04-1.16) |
|  | AZ: >4 weeks |  |  |  | 0.98 (0.93-1.02) |
|  | PF: >4 weeks |  |  |  | 1.09 (1.02-1.16) |

**Table S6**. Adjusted hazard ratios for infection (dosing interval)

| **Variable** | **Levels** | **Vaccine type** | **Number of individuals** | **Number of events** | **Adjusted hazard ratio** |
| --- | --- | --- | --- | --- | --- |
| **Unvaccinated** |  |  | 190202 | 26765 | Reference |
| **First dose** | 1-2 wks | ChAdOx-1 | 105580 | 5256 | 0.67 (0.6-0.75) |
|  |  | BNT162b2 | 47803 | 2934 | 0.68 (0.6-0.78) |
|  | 3 wks | ChAdOx-1 | 99045 | 1731 | 0.73 (0.63-0.85) |
|  |  | BNT162b2 | 44387 | 1031 | 0.56 (0.47-0.66) |
|  | 4 wks | ChAdOx-1 | 96744 | 921 | 0.58 (0.48-0.7) |
|  |  | BNT162b2 | 42583 | 633 | 0.48 (0.39-0.59) |
|  | 5 wks | ChAdOx-1 | 95140 | 654 | 0.59 (0.47-0.73) |
|  |  | BNT162b2 | 41521 | 403 | 0.44 (0.35-0.55) |
|  | 6-7 wks | ChAdOx-1 | 93718 | 642 | 0.5 (0.4-0.62) |
|  |  | BNT162b2 | 40877 | 548 | 0.52 (0.41-0.66) |
|  | 8-10 wks | ChAdOx-1 | 90634 | 347 | 0.53 (0.4-0.71) |
|  |  | BNT162b2 | 39539 | 468 | 0.8 (0.6-1.07) |
|  | 11+ wks | ChAdOx-1 | 57784 | 181 | 1.02 (0.73-1.43) |
|  |  | BNT162b2 | 28718 | 73 | 0.67 (0.46-0.96) |
| **Second dose** | 1-4 wks | ChAdOx-1 | 86845 | 119 | 0.44 (0.29-0.69) |
|  |  | BNT162b2 | 37328 | 120 | 0.49 (0.32-0.73) |
|  | 5-10 wks | ChAdOx-1 | 85615 | 134 | 0.61 (0.42-0.89) |
|  |  | BNT162b2 | 36785 | 45 | 0.43 (0.26-0.7) |
|  | 11-15 wks | ChAdOx-1 | 81979 | 327 | 0.52 (0.39-0.7) |
|  |  | BNT162b2 | 35430 | 57 | 0.32 (0.2-0.5) |
|  | 16-20 wks | ChAdOx-1 | 77764 | 1090 | 0.77 (0.62-0.96) |
|  |  | BNT162b2 | 34094 | 294 | 0.57 (0.41-0.81) |
|  | 21+ wks | ChAdOx-1 | 68221 | 1474 | 0.76 (0.6-0.95) |
|  |  | BNT162b2 | 31475 | 630 | 0.57 (0.44-0.74) |
| **Age group** | 65-69 years |  | 9346 | 1871 | Reference |
|  | 70-74 years |  | 16303 | 3619 | 1.15 (1.09-1.22) |
|  | 75-79 years |  | 23977 | 5626 | 1.26 (1.19-1.33) |
|  | 80-84 years |  | 37221 | 9247 | 1.36 (1.29-1.44) |
|  | 85-89 years |  | 48836 | 11999 | 1.36 (1.29-1.44) |
|  | 90+ years |  | 61450 | 14515 | 1.32 (1.25-1.4) |
| **Case rate** |  |  |  |  | 1.07 (1.05-1.08) |
| **Sex** | Female |  | 138662 | 32382 | Reference |
|  | Male |  | 58471 | 14495 | 1.14 (1.11-1.16) |
| **Relative deprivation** | 1 (least deprived) |  | 34669 | 8006 | Reference |
|  | 2 |  | 38828 | 9169 | 1.02 (0.93-1.12) |
|  | 3 |  | 41873 | 10118 | 1.06 (0.97-1.17) |
|  | 4 |  | 43117 | 9920 | 1.01 (0.92-1.11) |
|  | 5 (most deprived) |  | 38646 | 9664 | 1.13 (1.02-1.25) |
| **Scaled interval (weeks)** | AZ: 1-4 weeks |  |  |  | 1.07 (1.01-1.12) |
|  | PF: 1-4 weeks |  |  |  | 1.1 (1.04-1.16) |
|  | AZ: >4 weeks |  |  |  | 0.98 (0.93-1.02) |
|  | PF: >4 weeks |  |  |  | 1.09 (1.02-1.16) |

**Table S7**. Adjusted hazard ratios for death (dosing interval)

| **Variable** | **Levels** | **Vaccine type** | **Number of individuals** | **Number of events** | **Adjusted hazard ratio** |
| --- | --- | --- | --- | --- | --- |
| **Unvaccinated** |  |  | 190109 | 7425 | Reference |
| **First dose** | 1-2 wks | ChAdOx-1 | 105578 | 1364 | 0.58 (0.5-0.66) |
|  |  | BNT162b2 | 47801 | 761 | 0.6 (0.51-0.7) |
|  | 3-4 wks | ChAdOx-1 | 99324 | 485 | 0.49 (0.39-0.6) |
|  |  | BNT162b2 | 44556 | 327 | 0.35 (0.29-0.43) |
|  | 5-8 wks | ChAdOx-1 | 95636 | 178 | 0.37 (0.27-0.5) |
|  |  | BNT162b2 | 41783 | 169 | 0.34 (0.26-0.45) |
|  | 9+ wks | ChAdOx-1 | 86556 | 36 | 0.44 (0.27-0.71) |
|  |  | BNT162b2 | 37967 | 35 | 0.5 (0.32-0.78) |
| **Second dose** | 1-4 wks | ChAdOx-1 | 86843 | 9 | 0.17 (0.06-0.45) |
|  |  | BNT162b2 | 37325 | 9 | 0.16 (0.04-0.57) |
|  | 5-10 wks | ChAdOx-1 | 85610 | 10 | 0.19 (0.07-0.49) |
|  |  | BNT162b2 | 36784 | 5 | 0.21 (0.05-0.8) |
|  | 11-15 wks | ChAdOx-1 | 81971 | 39 | 0.23 (0.13-0.39) |
|  |  | BNT162b2 | 35428 | 4 | 0.09 (0.03-0.26) |
|  | 16-20 wks | ChAdOx-1 | 77717 | 155 | 0.39 (0.26-0.59) |
|  |  | BNT162b2 | 34087 | 38 | 0.27 (0.16-0.46) |
|  | 21+ wks | ChAdOx-1 | 64662 | 196 | 0.45 (0.3-0.68) |
|  |  | BNT162b2 | 30054 | 84 | 0.32 (0.2-0.5) |
| **Age group** | 65-69 years |  | 9332 | 242 | Reference |
|  | 70-74 years |  | 16287 | 593 | 1.5 (1.29-1.74) |
|  | 75-79 years |  | 23950 | 1115 | 2.06 (1.79-2.38) |
|  | 80-84 years |  | 37206 | 2064 | 2.62 (2.28-3.01) |
|  | 85-89 years |  | 48813 | 3044 | 3.08 (2.69-3.54) |
|  | 90+ years |  | 61431 | 4271 | 3.59 (3.13-4.12) |
| **Case rate** |  |  |  |  | 1.07 (1.05-1.09) |
| **Sex** | Female |  | 138586 | 6890 | Reference |
|  | Male |  | 58433 | 4439 | 1.84 (1.77-1.92) |
| **Relative deprivation** | 1 (least deprived) |  | 34647 | 1749 | Reference |
|  | 2 |  | 38799 | 2215 | 1.1 (0.96-1.26) |
|  | 3 |  | 41849 | 2487 | 1.17 (1.02-1.33) |
|  | 4 |  | 43095 | 2441 | 1.09 (0.96-1.25) |
|  | 5 (most deprived) |  | 38629 | 2437 | 1.24 (1.08-1.42) |
| **Scaled interval (weeks)** | 1-4 weeks after dose 2 AZ |  |  |  | 1.08 (0.95-1.23) |
|  | 1-4 weeks after dose 2 PF |  |  |  | 1.04 (0.86-1.25) |
|  | >4 weeks after dose 2 AZ |  |  |  | 0.99 (0.91-1.08) |
|  | >4 weeks after dose 2 PF |  |  |  | 1.03 (0.91-1.16) |

**Table S8**. Adjusted hazard ratios for infection (those with previous positive test)

| **Variable** | **Levels** | **Vaccine type** | **Number of individuals** | **Number of events** | **Adjusted hazard ratio** |
| --- | --- | --- | --- | --- | --- |
| **Unvaccinated** |  |  | 18216 | 344 | Reference |
| **First dose** | 1-2 wks | ChAdOx-1 | 11487 | 94 | 0.82 (0.59-1.14) |
|  |  | BNT162b2 | 5377 | 53 | 0.86 (0.62-1.2) |
|  | 3 wks | ChAdOx-1 | 11251 | 15 | 0.38 (0.2-0.7) |
|  |  | BNT162b2 | 5283 | 16 | 0.53 (0.31-0.93) |
|  | 4 wks | ChAdOx-1 | 11163 | 28 | 0.77 (0.44-1.36) |
|  |  | BNT162b2 | 5193 | 6 | 0.28 (0.12-0.65) |
|  | 5 wks | ChAdOx-1 | 11025 | 29 | 0.79 (0.45-1.38) |
|  |  | BNT162b2 | 5142 | 7 | 0.4 (0.17-0.93) |
|  | 6-7 wks | ChAdOx-1 | 10853 | 25 | 0.47 (0.25-0.87) |
|  |  | BNT162b2 | 5080 | 16 | 0.47 (0.25-0.9) |
|  | 8-10 wks | ChAdOx-1 | 10468 | 11 | 0.57 (0.22-1.47) |
|  |  | BNT162b2 | 4954 | 13 | 0.54 (0.25-1.15) |
|  | 11+ wks | ChAdOx-1 | 6703 | 9 | 1.12 (0.42-3.02) |
|  |  | BNT162b2 | 3617 | 3 | 0.72 (0.2-2.52) |
| **Second dose** | 1-4 wks | ChAdOx-1 | 10200 | 8 | 0.63 (0.27-1.5) |
|  |  | BNT162b2 | 4778 | 2 | 0.21 (0.05-0.85) |
|  | 5-10 wks | ChAdOx-1 | 10009 | 11 | 0.82 (0.29-2.32) |
|  |  | BNT162b2 | 4682 | 4 | 0.59 (0.18-1.96) |
|  | 11-15 wks | ChAdOx-1 | 9588 | 15 | 0.53 (0.2-1.4) |
|  |  | BNT162b2 | 4516 | 4 | 0.51 (0.14-1.85) |
|  | 16-20 wks | ChAdOx-1 | 9104 | 14 | 0.22 (0.09-0.51) |
|  |  | BNT162b2 | 4346 | 7 | 0.27 (0.1-0.74) |
|  | 21+ wks | ChAdOx-1 | 8043 | 29 | 0.35 (0.14-0.86) |
|  |  | BNT162b2 | 4060 | 10 | 0.2 (0.07-0.57) |
| **Age group** | 65-69 years |  | 787 | 35 | Reference |
|  | 70-74 years |  | 1601 | 57 | 0.82 (0.54-1.24) |
|  | 75-79 years |  | 2465 | 103 | 0.98 (0.67-1.45) |
|  | 80-84 years |  | 3855 | 146 | 0.91 (0.64-1.31) |
|  | 85-89 years |  | 4713 | 195 | 1.02 (0.71-1.46) |
|  | 90+ years |  | 5089 | 237 | 1.17 (0.82-1.68) |
| **Case rate** |  |  |  |  | 1.09 (1.05-1.13) |
| **Sex** | Female |  | 12615 | 511 | Reference |
|  | Male |  | 5895 | 262 | 1.17 (1-1.36) |
| **Relative deprivation** | 1 (least deprived) |  | 4041 | 181 | Reference |
|  | 2 |  | 3906 | 155 | 0.87 (0.7-1.09) |
|  | 3 |  | 3685 | 162 | 1 (0.79-1.25) |
|  | 4 |  | 3600 | 125 | 0.79 (0.63-1.01) |
|  | 5 (most deprived) |  | 3278 | 150 | 1.05 (0.84-1.33) |

**Figure S****6**. Adjusted hazard ratios for infection (residence type)


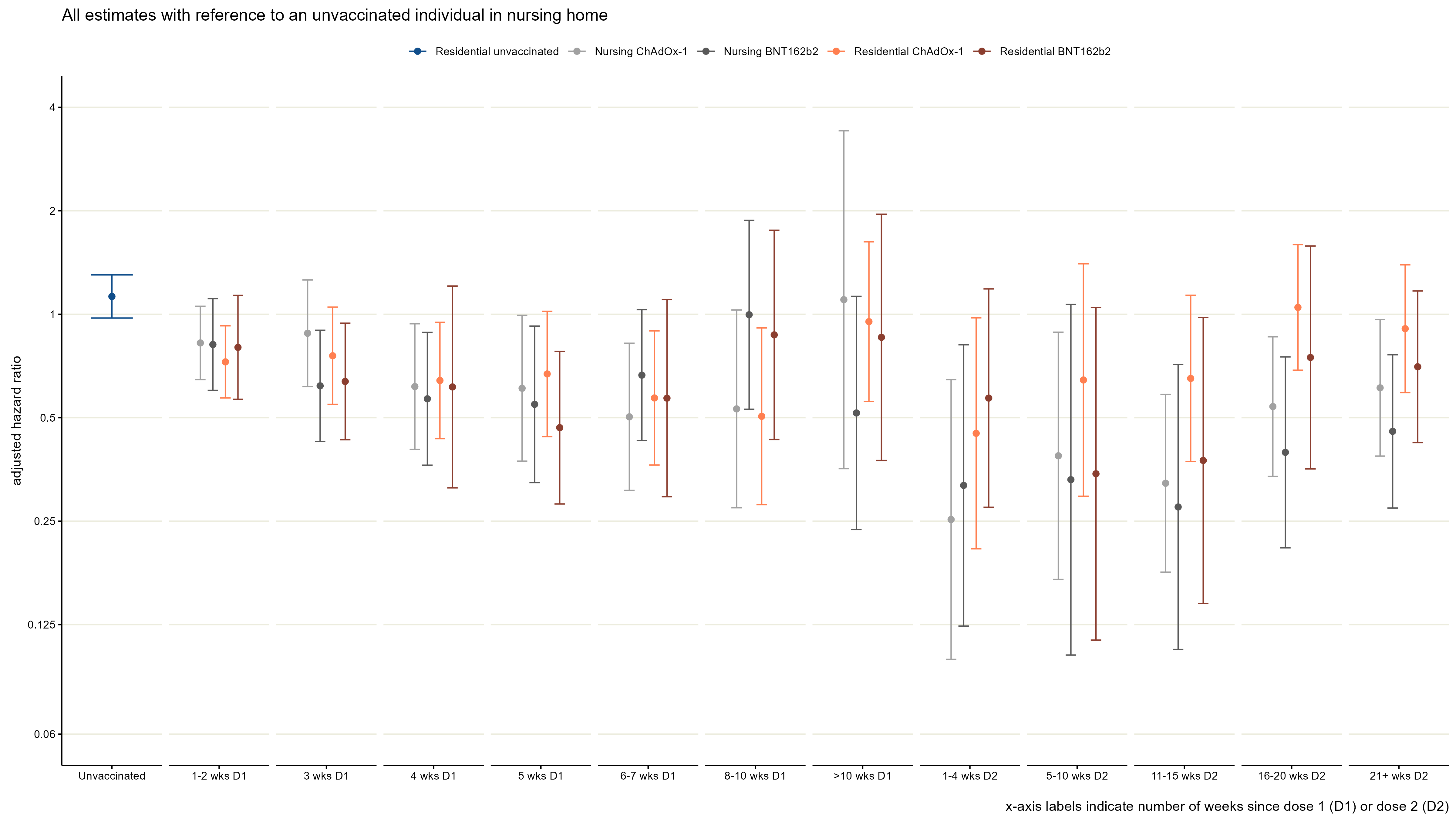


**Figure S****7**. Adjusted hazard ratios for death (residence type)


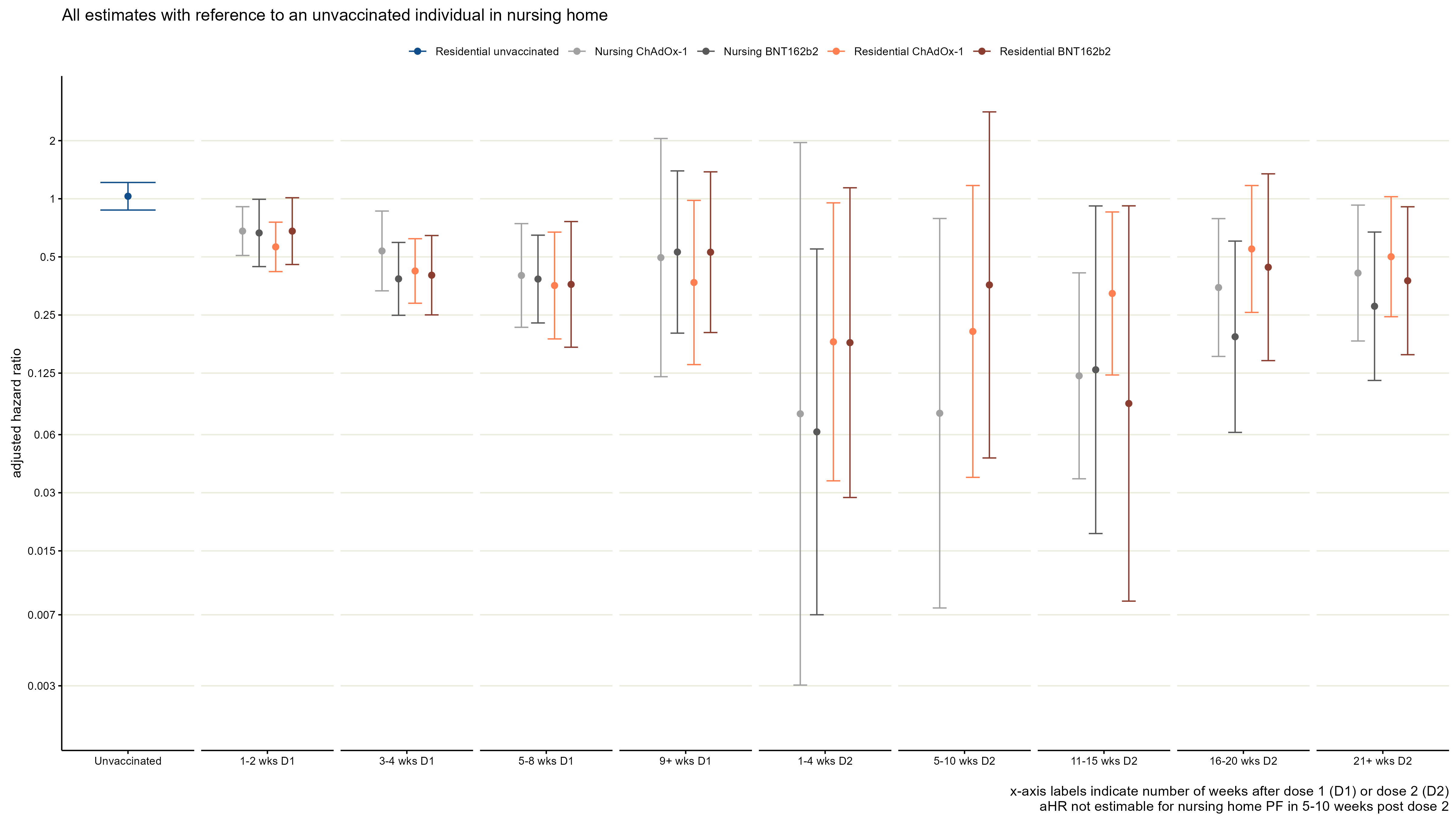

Supplement: aa-21-1873-File002_afac115 [file aa-21-1873-file002_afac115.docx]
